# The prevalence and clinical characteristics of diabetes mellitus in Chinese inpatients with chronic schizophrenia: a multicenter cross-sectional study

**DOI:** 10.7717/peerj.12553

**Published:** 2021-11-30

**Authors:** Yanni Wang, Lingyun Zeng, Lijuan Chen, Xin Zhou, Lijuan Huo, Tingwei Wang, Yongjie Zhou, Xiangyang Zhang

**Affiliations:** 1 Department of Maternal, Child and Adolescent Health, School of Public Health, Lanzhou, China; 2 Department of Psychiatric Rehabilitation, Shenzhen Kangning Hospital, Shenzhen, China; 3 School of Literature, Journalism & Communication, South-Central University for Nationalities, Wuhan, China; 4 Research Center for Psychological and Health Sciences, China University of Geosciences, Wuhan, China; 5 Department of Psychiatry, Affiliated Brain Hospital of Guangzhou Medical University, Guangzhou, China; 6 CAS Key Laboratory of Mental Health, Institute of Psychology, Chinese Academy of Sciences, Beijing, China

**Keywords:** Schizophrenia, Diabetes mellitus, Prevalence, China, Clinical characteristics

## Abstract

**Background:**

Diabetes mellitus (DM) is common among patients with schizophrenia. However, information on patients comorbid DM and schizophrenia is limited in China. The present study investigated the prevalence of DM and its clinical characteristics in Chinese inpatients with chronic schizophrenia.

**Methods:**

A cross-sectional study was performed in Chinese inpatients with chronic schizophrenia. Diagnosis of Diabetes was established using World Health Organization diagnostic criteria for diabetes mellitus (persistent fasting glucose levels ≥ 126 mg/dl or 2-h plasma glucose ≥ 200 mg/dL after a 75-g Oral Glucose Tolerance Test). Patients were also measured height, weight, waist circumference, hip circumference, triglyceride level, and cholesterol level. Patients’ psychiatric symptoms were measured by the Positive and Negative Syndrome Scale (PANSS). Binary logistic regression analysis was performed to examine the associated demographic and clinical variables in chronic schizophrenia.

**Results:**

A total of 988 inpatients (64.6% male, average age of 47.19 ± 12.55) was recruited. The prevalence of DM in Chinese patients with chronic schizophrenia was 13.8% (95% CI [11.6–15.9]%). Logistic regression analysis showed that overweight (OR = 1.90, 95% CI [1.20–3.03], *p* = 0.006), obesity (OR = 1.85, 95% CI [1.07–3.21], *p* = 0.028), comorbid hypertension (OR = 2.14, 95% CI [1.34–3.42], *p* = 0.002), and course of schizophrenia (OR = 1.03, 95% CI [1.01–1.06], *p* = 0.040) were significantly associated with the DM risk in patients with schizophrenia.

**Conclusion:**

The findings indicated that diabetes mellitus was non-negligible in patients with chronic schizophrenia. Patients with schizophrenia should be regularly monitored for DM. Overweight/obesity, long duration of schizophrenia, and comorbid hypertension possibly were risk factors for diabetes.

## Introduction

Diabetes mellitus (DM) have a high prevalence in patients with schizophrenia. The increase in the incidence of DM and related premature mortality has become a major concern for patients with schizophrenia. A recent meta-analysis, including 25 studies involving 145,718 patients, showed that the global prevalence of DM in schizophrenia was 10.75% (Stubbs et al., 2015). The incidence of diabetes in schizophrenia is estimated to be 1.5–3 times higher than that in the general population (Mitchell et al., 2013), indicating that patients with schizophrenia have a higher risk of developing DM than the general population (Vancampfort et al., 2016; Vancampfort et al., 2015). In a large population-based cohort study (Ribe et al., 2014), the authors evaluated the cumulative risks of death after being diagnosed with DM in people with severe mental illness. They found that people with schizophrenia or schizo-affective disorders combined with diabetes have a three to four times higher death risk than the general population. The interaction between DM and severe mental illness in terms of mortality is much greater than their independent existence.

It is complex and multifactorial for the increased incidence of diabetes in patients with schizophrenia. Previous studies have observed overlapping genetic variations between schizophrenia and DM (Hackinger et al., 2018). In addition to the influence of genetic factors, environmental factors play an essential role in the etiology of diabetes in schizophrenia. A growing body of evidence suggests that the use of antipsychotics, particularly atypical antipsychotics, may increase the risk of developing diabetes, and mentioned a dose-dependent increase in the risk of diabetes in those taking atypical antipsychotics (Holt, 2019). Additionally, schizophrenia patients tend to have an unhealthy lifestyle, characterized by poor dietary choices, lack of exercise and smoking (Sugai et al., 2016). Those are considered an important factor related to the high prevalence of DM.

Some researchers documented the prevalence and risk factors of DM in Chinese patients with schizophrenia. An earlier case-control study showed that the prevalence of DM was 22.3% among Chinese schizophrenia patients who received long-term clozapine treatment (Zhang et al., 2011). Yang et al. (2020) reported a prevalence of 11.63% of DM among Chinese inpatients with schizophrenia, and found that inpatients living in urban, widowed and old age had a high risk for DM. A recent cross-sectional study from one psychiatric hospital reported that the prevalence of DM in patients with schizophrenia was 12.5%, and found that age, high body mass index (BMI) and triglyceride levels were associated with diabetes in schizophrenia inpatients (Huo et al., 2020). However, these studies differed in the prevalence and risk factors of DM with schizophrenia. In the current study, we conducted a cross-sectional multicenter survey to identify the prevalence and clinical features of DM in hospitalized patients with chronic schizophrenia in China.

### Methods

### Ethical considerations

The protocol of this study was approved by the ethics committee of the Institutional Review Board (IRB 18120501) of the Institute of Psychology, Chinese Academy of Sciences. All participants provided written informed consent to take part in the study.

### Participants

This was a case series study. All the participants were selected from 4 psychiatric hospitals (Wuhan Youfu Hospital, Wuhan Mental Health Center, Wuhan Xinzhou Mental Health Center, and Guangzhou Hui’ai Hospital). Han Chinese patients were recruited in this study if they met the following criteria: (1) diagnosed with schizophrenia according to the Structured Clinical Interview for DSM-IV (SCID) (Phillips et al., 2007) and conducted by two psychiatrists. (2) aged ≥18 years old; (3) had multiple previous admissions to hospital for schizophrenia (≥2 times), and (4) the patients’ duration of illness was more than two years (Chen, 2002). Patients were excluded according to the following criteria: (1) pregnancy or lactation, (2) with severe physical diseases, (3) a history of mental retardation. A total of 1,090 patients with schizophrenia were recruited. Ninety participants denied consent for participation. Twelve participants did not complete the questionnaire. Finally, a total of 988 Han Chinese inpatients were recruited in this study.

### Demographic characteristics

Personal data on age, sex, education, and marital status were collected through a self-designed questionnaire. The age of onset of schizophrenia, the total length of hospitalization, duration of illness, psychotropic drugs (type and dose) and physical comorbidities were collected through medical records and clinical interviews.

### Clinical measures

Diagnosis of DM was based on inpatients’ medical records and their current medication use. Diabetes mellitus was diagnosed according to World Health Organization diagnostic criteria. Scores of positive, negative and disorganized symptoms were calculated by the Positive and Negative Syndrome Scale (PANSS) (Kay, Fiszbein & Opler, 1987).

All the measurement tools used in this study were verified in China (Si et al., 2004; Zang et al., 2008). In order to ensure the consistency and reliability of the scores, all raters participated in a training course on the use of the scales before the start of the study. All the raters who scored the scales were blind to the clinical condition of the patients.

### Blood glucose and lipids

Blood samples from all patients were collected between 7 am and 9 am. Triglyceride and cholesterol were measured using in-hospital laboratory facilities.

### Anthropometric assessments

Height, weight, waist circumference and hip circumference were measured by the standardized method. BMI and waist-hip ratio were calculated to describe the physical characteristics of the patients. We adopted the definition of BMI cut-off values of 24.0–27.9 kg/m^2^ for overweight and ≥ 28.0 kg/m^2^ for obesity recommended by the Working Group on Obesity in China (WGOC) (Zhou, 2002)

### Statistical analysis

The socio-demographic data and clinical characteristics of patients with and without DM were described. Kolmogorov–Smirnov one-sample test was calculated to examine whether each parameter obeyed the normal distribution. Analysis of variance (ANOVA) for continuous variables, chi-square test for categorical variables, and Mann–Whitney *U* test for non-normally distributed variables were utilized to compare differences in the characteristics of the two groups (schizophrenia with and without DM). Multiple regression analysis was used to identify the significant factor related to DM in patients with chronic schizophrenia by calculating odds ratio (OR) and 95% confidence interval (CI). The socio-demographic and clinical characteristics were included in the multiple regression analysis according to results of the univariate analysis.

All analyses were performed using SPSS version 22.0 software (SPSS Inc., Chicago, IL, USA). All* * tests were two-tailed, and statistical significance was defined at *p* < 0.05.

## Results

### Demography of participants

A total of 988 patients were recruited, including 638 men and 350 women. The average age of patients was 47.19 ± 12.55 years. The average course of the disease was 21.71 ± 4.70 years.

The prevalence of DM was 13.8% (95% CI [11.6–15.9%]) in our sample. Table 1 shows the demographic characteristics of inpatients with (*n* = 136) and without DM (*n* = 852). There were no significant differences in sex, marital status between the two groups (all *p* > 0.05). DM patients were older than non-DM patients (55.36 ± 9.06 *vs.* 45.88 ± 12.53, *p* < 0.001, Table 1).

**Table 1:** Demographic profile of schizophrenia patients with and without diabetes mellitus (DM).

Characteristics	Total patients (*N* = 988)	Patients with DM (*N* = 136)	Patients without DM (*N* = 852)	*F*/*χ*^2^	*p*-value
Hospitals, N(%)				20.17	0.001
Wuhan Youfu Hospital	174 (17.6)	16 (11.9)	158 (18.5)		
Wuhan Mental Health Center	138 (14.0)	24 (17.6)	114 (13.4)		
Wuhan Xinzhou Mental Health Center	328 (33.2)	24 (17.6)	304 (35.7)		
Guangzhou Huiai Hospital	348 (35.2)	72 (52.9)	276 (32.4)		
Age(yrs), M ± SD	47.19 ± 12.55	55.36 ± 9.06	45.88 ± 12.53	33.65	<0.001
Sex, N(%)				2.90	0.089
Male	638 (64.6)	79 (58.1)	559 (65.6)		
Female	350 (35.4)	57 (41.9)	293 (34.4)		
Marital status, N(%)				0.93	0.627
Married	228 (23.1)	28 (20.7)	200 (23.5)		
Never married	583 (59.2)	85 (63.0)	498 (58.6)		
Divorced or widowed	174 (17.7)	22 (16.3)	152 (17.9)		
Education (yrs), M ± SD	9.19 ± 3.21	9.12 ± 3.21	9.59 ± 3.23	2.50	0.114

### The clinical characteristics of chronic schizophrenia with *vs.* without MD

As Table 2 showed, compared with non-DM patients, DM patients had a larger waist circumference and waist-hip ratio (*F* = 12.86, *p* < 0.001; *F* = 3.93, *p* =0.048). Furthermore, DM patients were more likely to be overweight or obese (*F* = 6.23, *p* =0.045). There were significant differences between the two groups in terms of the duration of schizophrenia, the total length of hospitalization, and the history of hypertension and cerebrovascular disease (all *p* < 0.05). In addition, DM patients had a higher dose of antipsychotic drugs than non-DM patients (*p* < 0.001). More DM patients took sedatives than non-DM patients (24.3% *vs.* 15.5%, *p* = 0.039). There were no significant differences in PANSS scores (total score, positive symptom subscore, negative symptom subscore, and general psychopathology subscore).

**Table 2:** Clinical characteristics of schizophrenia patients with and without diabetes mellitus (DM).

	Total patients (*N* = 988)	Patients with DM (*N* = 136)	Patients without DM (*N* = 852)	*F*/*χ*^2^/*Z*	*p*-value
PANSS scores (M ± SD)					
Total score	78.57 ± 17.41	78.33 ± 15.48	78.61 ± 17.72	0.03	0.861
Positive symptom subscore	16.28 ± 5.36	16.13 ± 5.20	16.31 ± 5.39	0.12	0.729
Negative symptom subscore	21.83 ± 7.02	21.90 ± 5.98	21.82 ± 7.18	0.39	0.530
General psychopathology subscore	40.45 ± 8.70	40.30 ± 7.72	40.48 ± 8.86	0.05	0.820
Age at onset of schizophrenia (yrs), M ± SD	25.48 ± 8.01	25.32 ± 7.97	26.47 ± 8.27	2.43	0.119
Duration of schizophrenia (yrs), M ± SD	21.73 ± 11.73	29.10 ± 9.80	20.58 ± 11.73	64.02	<0.001
Total length of hospitalization(month), Median (IQR)	48.0 (12.0,125.5)	92.0 (12.0,192.0)	43.0 (12.0,120.0)	−3.15	0.006
Medical comorbidity					
Hypertension, N(%)	149 (15.1)	47 (34.5)	102 (12.0)	46.72	<0.001
Cerebrovascular disease, N(%)	20 (2.0)	10 (7.4)	10 (1.2)	22.15	<0.001
BMI, N(%)				7.95	0.019
<24.0 kg/m^2^	467 (47.4)	49 (36.3)	418 (49.1)		
24.0–27.9kg/m^2^	326 (33.0)	56 (41.5)	270 (31.7)		
≥28.0 kg/m^2^	193 (19.6)	30 (22.2)	163 (19.2)		
Smoke behavior, N(%)				0.29	0.863
Daily smoker	265 (28.7)	37 (29.4)	228 (28.6)		
Former smoker	124 (13.4)	15 (11.9)	109 (13.7)		
Ever smoker	534 (57.9)	74 (58.7)	460 (57.7)		
Waist-hip ratio, M ± SD	0.93 ± 0.09	0.95 ± 0.07	0.93 ± 0.09	3.93	0.047
Fasting plasma glucose (mmol/L), M ± SD	5.16 ± 2.33	6.24 ± 2.70	5.01 ± 2.23	37.47	<0.001
Cholesterol (mmol/L), M ± SD	4.31 ± 0.96	4.31 ± 0.98	4.30 ± 0.96	0.02	0.983
Triglyceride (mmol/L), M ±SD	1.62 ± 1.12	1.52 ± 1.04	1.66 ± 1.15	−1.35	0.177
Antipsychotic medication, N(%)				2.49	0.109
Atypical	982 (99.4)	136 (100.0)	846 (99.3)		
Typical	6 (0.6)	0 (0.0)	6 (0.7)		
Antipsychotic dose (mg/day, CPZ equivalents), Median(IQR)	250 (145,400)	345 (200,525)	240 (125,400)	−3.98	<0.001
Sedatives usage, N(%)				4.52	0.039
Yes	165 (18.2)	33 (24.3)	132 (15.5)		
No	823 (81.8)	103 (75.7)	720 (84.5)		

**Notes.**

PANSS, Positive and negative syndrome scale; CPZ equivalents, Calculation by chlorpromazine equivalents.

### Associated factors of diabetes mellitus in patients with chronic schizophrenia

The results of multiple logistic regression analysis showed that overweight (OR = 1.90, 95% CI [1.20–3.03], *p* = 0.006), obesity (OR = 1.85, 95% CI [1.07–3.21], *p* = 0.028), comorbid hypertension (OR = 2.14, 95% CI [1.34–3.42], *p* = 0.002), and course of schizophrenia (OR = 1.03, 95% CI [1.01–1.06], *p* = 0.040) were significantly associated with DM in patients with chronic schizophrenia after adjusting for the different psychiatric hospitals (Table 3).

**Table 3:** Logistic regression analyses for factors related to diabetes mellitus in schizophrenia.

Variables	*B*	*S.E*	*p*	OR (95% CI)^a^
Age	0.03	0.15	0.050	1.03 (1.00–1.06)
BMI				
<24.0 kg/m^2^				reference
24.0–27.9kg/m^2^	0.65	0.24	0.006	1.90 (1.20–3.03)
≥28.0 kg/m^2^	0.62	0.28	0.028	1.85 (1.07–3.21)
Course of schizophrenia	0.03	0.01	0.040	1.03 (1.01–1.06)
Medical history of hypertension	0.76	0.24	0.002	2.14 (1.34–3.42)

**Notes.**

a The ORs and 95% CIs were calculated by logistic regression analysis adjusted for the different psychiatric hospitals.

## Discussion

To the best of our knowledge, this is the first large-scale multicenter study in China to investigate the epidemiology and clinical correlates of DM in patients with chronic schizophrenia. The main findings of this study were: (1) the prevalence of DM in patients with chronic schizophrenia was 13.8% (95% CI [11.6–15.9]%), (2) other medical comorbidities (hypertension, cerebrovascular disease), physical condition (high BMI and waist-to-hip ratio), and history of schizophrenia (course of schizophrenia, total length of hospitalization) may be associated with DM in patients with chronic schizophrenia, and (3) logistic regression analysis showed that overweight/obesity, duration of schizophrenia, and comorbid hypertension were significantly associated with DM in patients with chronic schizophrenia.

The prevalence of DM in people with schizophrenia varies markedly worldwide, ranging from 1.26% to 50% (Ward & Druss, 2015). Our findings showed that the prevalence of DM in the inpatients with chronic schizophrenia was 13.8%, which was higher than the prevalence of 10.9% in the general Chinese population (Wang et al., 2017). This result is consistent with previous studies (Schoepf et al., 2012; Stubbs et al., 2015). Some theories have attempted to explain why patients with schizophrenia are prone to diabetes. Previous studies have shown that schizophrenia and type 2 diabetes may have the same genetic susceptibility (Lin & Shuldiner, 2010). Some genetic studies of DM and schizophrenia have also revealed many overlapping risk genes, such as those involved in calcium, adipocytokine, insulin, and AKT signaling pathways (Hackinger et al., 2018; Sargazi et al., 2020). At the same time, the literature confirmed a causal relationship between genetic susceptibility to high fasting insulin levels and increased risk of schizophrenia, while schizophrenia-related variants have no effect on fasting insulin levels (Li et al., 2018).

The relation between DM and the use of atypical antipsychotics has been widely described. A large meta-analysis of 438,245 people found that prior to the use of psychotropic drugs, the prevalence of type 2 diabetes was 2.9%, while in patients with severe mental illness treated with antipsychotic medications, it increased to 11.3% (Vancampfort et al., 2016). There is growing evidence that most atypical antipsychotics, especially olanzapine and clozapine, are associated with a high prevalence of diabetes (Holt, 2019). However, in this study, we did not find that atypical antipsychotics were an independent risk of DM in patients with schizophrenia, which is in accordance with the results of the previous study from our research group (Huo et al., 2020) and Rawat et al. (2018). In addition to antipsychotics, sedatives are frequently prescribed in patients with schizophrenia and are widely used to reduce the increased risk of impulsive violent behaviors (Kumari et al., 2009), catatonia (Zaman, Gibson & Walcott, 2019), and insomnia (Chiu et al., 2016). In a Dutch pharmacy database study, 60,516 individuals were followed up after the first prescription of sedatives. Compared with people who did not take sedatives, people who took sedatives had a significantly higher incidence rate of diabetes, with an incidence rate of 3.4 (Knol et al., 2009). Our findings showed a difference in sedative drug use of sedatives usage between the diabetic and non-diabetic patients with schizophrenia. However, this difference may be due to different antipsychotic drug choices by clinicians.

Compared with non-DM patients, DM patients had a longer duration of schizophrenia. A recent meta-analysis found that patients with multiple episodes of psychosis had a higher prevalence of diabetes than patients with first-episode (Vancampfort et al., 2016). Another meta-analysis showed that the course of schizophrenia itself was a risk factor for metabolic syndrome (Vancampfort et al., 2015). These studies further proved that the longer the duration of schizophrenia, the higher the risk of developing DM.

Further, we found significant associations between DM and overweight/obesity and hypertension. These associations are consistent with the previous literature (Holt, 2019; Huo et al., 2020; Mookhoek et al., 2011). Obesity is a significant risk factor for the development of type 2 diabetes (Tao, Shi & Zhao, 2015). The literature reported that the rates of overweight and obesity in patients with severe mental illness had increased by 2–3 times (Holt & Peveler, 2009). Overweight and obesity usually occur in the early stages of schizophrenia (Holt, 2019). Kahn et al. (2008) observed a substantial and rapid weight gain in patients with first-episode schizophrenia within 6–8 weeks after receiving antipsychotic drugs. A systematic review reported that patients with chronic schizophrenia who received long-term treatment gained an average weight of 5.6 kg (Citrome et al., 2011). Weight gain may be due to the use of antipsychotics (Holt, 2019). A recent meta-analysis demonstrated that people with schizophrenia had very high levels of sedentary behavior (Stubbs et al., 2016). Moreover, unhealthy food choices, social deprivation, and disease-specific mechanisms contribute to obesity in patients with schizophrenia (Holt, 2019). As a result, the high risk of overweight and obesity in patients with schizophrenia increases the likelihood of developing DM.

Previous studies mentioned that schizophrenia patients with DM may have more severe positive symptoms and negative symptoms than patients without DM (Bora, Akdede & Alptekin, 2017; Han et al., 2013; Huo et al., 2020). However, the current study found no association between the presence of DM and psychiatric symptoms in inpatients with chronic schizophrenia.

Our findings suggested that diabetes is not negligible in patients with chronic schizophrenia, and might have potential implications for managing patients with schizophrenia. Clinicians should be alert of diabetes mellitus in patients with a long course of schizophrenia. Blood glucose testing should be listed as a routine examination of schizophrenia for early detection and consequent treatment (Sugai et al., 2016). However, other important factors, such as poor dietary choices and reduced physical activity, may play an essential role in DM (Tao, Shi & Zhao, 2015). Insights provided by further research in these areas may better help improve for the management of patients with schizophrenia.

However, several limitations should be noted in this study. First, its cross-sectional nature limits the interpretation of the causal relationship between DM and risk factors in patients with schizophrenia. Future research may focus on longitudinal studies to confirm causality. Second, since our sample was composed of inpatients treated in psychiatric hospitals, the results may not necessarily be generalized to outpatients with schizophrenia. Third, lifestyle, such as dietary choices, excise habit, plays an important role in the etiology of DM, which unfortunately was not measured in this study.

In conclusion, this study shows that the prevalence of DM in patients with chronic schizophrenia is 13.8% in Chinese Han population, suggesting that patients with schizophrenia should be regarded as a high-risk group for diabetes. Moreover, our data show that patients with schizophrenia coexisting with DM are more likely to overweight/obesity and have hypertension. Importantly, the duration of schizophrenia may be a risk factor for diabetes.

## Supplemental Information

10.7717/peerj.12553/supp-1 Supplemental Information 1 Data of schizophrenia patients

## Additional Information and Declarations

### Data Availability

The following information was supplied regarding data availability: The raw measurements are available in the Supplemental File.
